# Resilience, Social Support, and Coping Strategies in Mitigating Psychological Distress Among Rural African American Women in South Carolina During the COVID-19 Pandemic: A Mediation Moderation Analysis

**DOI:** 10.1007/s40615-025-02645-w

**Published:** 2025-09-19

**Authors:** Atena Pasha, Shan Qiao, Sara Wilcox, Bankole Olatosi, Xiaoming Li

**Affiliations:** 1 Department of Health Promotion, Education and Behavior, Arnold School of Public Health, University of South Carolina, Columbia, SC USA; 2 South Carolina SmartState Center for Healthcare Quality, University of South Carolina, Columbia, SC USA; 3 Department of Exercise Science, Arnold School of Public Health, University of South Carolina, Columbia, SC USA; 4 Prevention Research Center, University of South Carolina, Columbia, SC USA; 5 Department of Health Services Policy and Management, Arnold School of Public Health, University of South Carolina, Columbia, SC USA

**Keywords:** African American women, Resilience, Social support, Coping, Mental health

## Abstract

**Background:**

Marginalization of intersecting racial, sex, and rural identities heightens the vulnerability of African American women in rural South Carolina (SC) during the COVID-19 pandemic, leading to psychological distress. Social support, positive coping, and resilience may serve as protective factors, enhancing individuals' ability to cope with stress.

**Methods:**

A cross-sectional online survey of 328 African American women (Mean age = 48.03 ± 16.75) in rural SC from January to February 2024 was conducted. Structural equation modeling was used to test direct, indirect, and total effects of social support on PTSD and hopelessness, mediated by resilience and coping. We also examined moderation by age and caretaker status.

**Results:**

All model fit indices were acceptable (CFI = 0.99, TLI = 0.98, RMSEA = 0.034, SRMR = 0.031, χ^2^ = 11.02, p = 0.2). Social support was positively associated with resilience (β = 0.463, *p* < 0.001), while resilience was negatively correlated with negative coping (β = -0.329, *p* < 0.001). Negative coping was linked to PTSD (β = 0.325, *p* < 0.001) and hopelessness (β = 0.321, *p* < 0.001). Resilience and positive coping mitigated hopelessness, and social support offered protection against PTSD. Resilience mediated the relationship between social support and hopelessness (β = -0.197, *p* < 0.001). The moderation analysis showed that single caretaker status negatively moderated the association between social support and resilience (β = -0.227, p = 0.006), while age positively moderated the association between resilience and hopelessness (β = 0.162, *p* = 0.001). Findings revealed that greater social support was associated with higher resilience and increased use of positive coping strategies. These, in turn, were linked to lower levels of hopelessness and PTSD symptoms. Moderation analysis indicated that the protective effect of social support is stronger among older women but weaker among single caretakers.

**Conclusions:**

Findings highlight the need for strategies tackling structural barriers to mental health equity in rural African American communities while considering individual factors like age and caretaker status, ensuring social support interventions yield positive outcomes.

## Introduction

In the United States (US), mental health disparities persist among women, who are more likely to experience psychological distress such as depression, hopelessness, and post-traumatic stress disorder (PTSD) [1–3]. In addition to factors such as experiences of abuse, trauma, and self-esteem, the prevalence of psychological distress in African American women may be influenced by sociocultural factors such as the Strong Black Woman (SBW) schema, which emphasizes emotional restraint and self-reliance. [3, 4]. The COVID-19 pandemic further increased social inequalities among African Americans, where they experienced more educational [5] and employment barriers, such as job or wage loss, compared to white individuals due to the pandemic [6] and experienced higher levels of food insecurity and rent or mortgage defaults than other racial and ethnic groups [7].

These disparities can also be understood through the minority stress framework, which posits that individuals with marginalized identities, such as racial, ethnic, gender, or sexual minorities, are exposed to chronic, identity-related stressors, including discrimination, stigma, and microaggressions [8]. These stressors exist above and beyond general life stress and can accumulate over time to erode mental and physical health. This framework also explains how the interplay of structural racism, sexism, and societal role expectations among African American women contributes to heightened levels of psychological distress, particularly hopelessness and PTSD, which have been identified as highly prevalent and debilitating among this population [9–12].

On the other hand, individual factors such as age, socioeconomic status, and caretaker responsibilities might moderate the relationship between stress and protective factors such as social support and resilience [8, 13–16]. For instance, older African Americans are likely to report more hopelessness compared to the young [17]. Besides hopelessness, resilience levels among the elderly are less than those of the young [18]. In African American culture, women often feel obligated to fulfill multiple roles, acting as both financial providers and caretakers, who need to develop the ability to independently support their families [4]. This socio-historically and culturally constructed concept of the SBW emphasizes resilience and inner strength, but may limit opportunities for self-awareness and self-care [19]. Experiencing chronic stressors can activate psychosocial processes that diminish an individual's ability to cope with stress. This can increase their vulnerability to psychological distress, particularly symptoms related to trauma, such as PTSD. It may also lead to a sense of hopelessness, which reflects a reduced outlook on the future and a loss of optimism [20, 21]. As a result, African American women might struggle to express their emotions, weaknesses, and needs [4].

Moreover, in southern states, structural racism and oppression led to poor health infrastructure, biased and suboptimal care, and limited access to care, such as a lack of health insurance and geographic maldistribution of health care services [17, 22–27]. However, during the COVID-19 pandemic, despite the many burdens and challenges that rural African American women have experienced, many have demonstrated more strength and resilience to overcome challenges and manage critical resources [18, 28]. Resilience is a dynamic and multidimensional construct that involves a two-way interaction between individuals and their environment and can be fostered through a range of protective factors, such as social support [29–33].

Social support is the process of social interaction in relationships that enhances belonging, coping, and respect through the exchange of physical or psychosocial resources [34]. Social support helps people cope with traumatic experiences and has been studied as one of the most robust predictors of PTSD symptoms [35, 36]. In African Americans, belonging support, which is a component of social support, meaning feeling valued and respected in the community, has been shown to negatively predict PTSD [37, 38]. Additionally, social support is negatively associated with hopelessness among African Americans, which is a risk factor for major depression, cardiovascular disease, and all-cause mortality [39].

Stress management and coping styles among African American women also differ from other ethnic groups of women in the US [40]. Coping refers to strategies individuals use to manage stressors in their environment [41]. Some strategies generally regarded as positive, such as positive reframing, acceptance, humor, active coping, and seeking social support, which have been associated with lower psychological distress [42–44]. Conversely, negative coping strategies, such as self-blame, venting, behavioral disengagement, self-distraction, and substance use coping, have been linked with more distress, including anxiety, depression, and PTSD [31, 32]. Among this population, coping is highly dependent on the social support they receive, which not only influences their coping skills but also contributes to their resilience-building [45–47]. Previous research indicated that African American women who use negative strategies to cope with psychological distress, rather than expressing emotions to individuals in peer groups or communities, may exacerbate mental health consequences without adequate support [29, 48, 49].

The study of the impact of COVID-19 on African Americans in Southern states is critical, as these regions were among the last to be locked down and the first to be reopened and had fewer pandemic-related restrictions compared to other areas of the US. Furthermore, the rural South is where systemic racism and oppression have led to elevated poverty rates and health disparities for Black African Americans [12, 27, 50–53]. Empirical data are particularly limited on the needs and challenges of African American women in rural SC, how they cope with challenges, and facilitators of resilience [54–57]. Understanding the links between social support and mental health in African American women during COVID-19 can help reduce mental health challenges and inform preparedness for future public health emergencies.

Guided by the Transactional Model of Stress and Coping [41] and Pearlin’s Stress Process Model [58], this study aimed to provide a comprehensive framework for examining how external stressors, coping resources, and sociocultural context interact to shape mental health outcomes. The Transactional Model conceptualizes stress as a dynamic interaction between individuals and their environment, emphasizing how people cognitively appraise stressors and utilize coping strategies to manage them, which is influenced by access to social support and personal resources such as resilience [59]. Pearlin’s Stress Process Model builds upon this by putting stress within a broader sociological context. It highlights how structural factors (e.g., race, class, gender roles) shape exposure to stressors and access to coping resources over time [58]. This model distinguishes between external resources (e.g., social support systems) and internal resources (e.g., psychological resilience and coping efficacy), making it particularly relevant to studies involving marginalized populations [60].

**Fig. 1:**
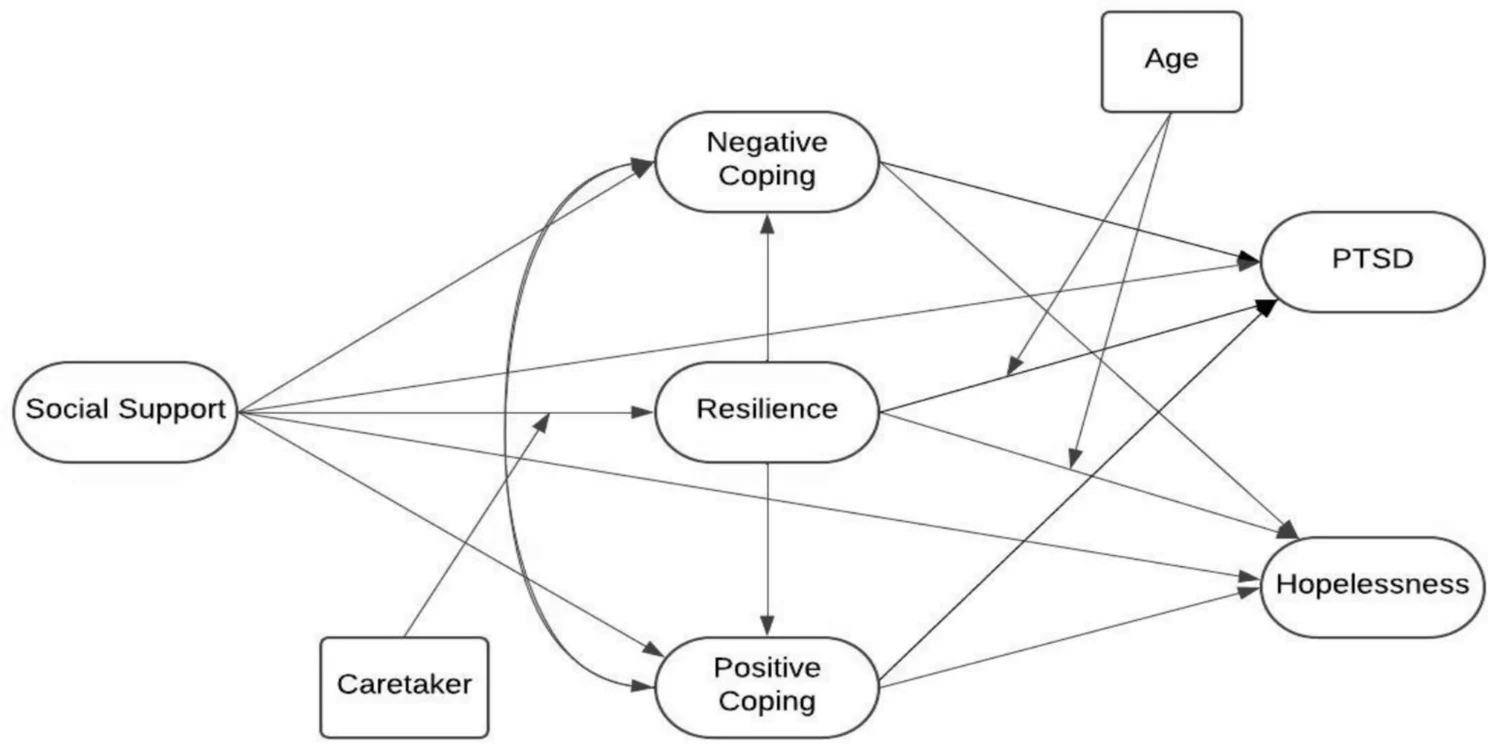
Conceptual Model of the Impact of Social Support on PTSD and Hopelessness through Resilience and Coping Strategies

Guided by these frameworks, this study examined how perceived social support influences psychological distress, measured by symptoms of PTSD and hopelessness, among African American women in rural SC. Hopelessness and PTSD, reflecting both future-oriented despair and trauma-related symptoms, serve as important indicators of psychological distress during the COVID-19 pandemic. Research demonstrates that African American women in the rural South confront significant challenges, facing heightened risks of trauma exposure and persistent uncertainty about the future [12, 61]. Understanding these experiences is vital to empowering this resilient population in their journey toward psychological well-being. In this regard, we test whether the relationship between social support (exposure) and psychological distress (outcomes) is mediated by two internal resources (i.e., resilience and coping strategies). These mediators are modeled in parallel, allowing us to evaluate their unique contributions to mental health outcomes. Additionally, we explore whether the strength of these relationships is moderated by age and caregiver status, two demographic characteristics that may shape the effectiveness of social support and internal coping processes. We expect that women who perceive higher levels of social support are less likely to experience psychological distress. Additionally, we hypothesize that coping and resilience serve as mediators in the relationship between social support and psychological distress among African American women in rural SC. Furthermore, we believe that these moderating factors may either strengthen or weaken the protective effects of social support and resilience on psychological distress (see Fig. 1).

## Methods

### Participants

A cross-sectional online survey was conducted in rural SC from January to February 2024. The specific study protocol has been reported in another paper [12]. To be eligible to participate in this survey, individuals were required to be 18 years of age or older, identify as an African American woman, and live in rural SC. Participants were identified and recruited through cluster-based sampling approach conducted by trained community health workers (CHWs) and community-engaged researchers across 18 rural counties in SC. The CHWs lived in the communities where they conducted recruitment and used their local knowledge to engage potential participants who met the study’s inclusion criteria. CHWs received training to implement recruitment strategies that emphasized inclusivity and representation, ensuring that participants reflected the diversity of the broader rural community. Electronic consent was provided online. Upon completing the survey, which took up to 60 min to complete, each participant received a $25 gift card. Participation in this study was confidential, and the survey did not collect any identifiable personal information.

A total of 360 individuals completed the survey, of whom 32 were excluded because they did not meet all inclusion criteria or had most of their survey uncompleted. Finally, 328 respondents were included in the current study. According to widely accepted Structural Equation Modeling (SEM) guidelines, a minimum of 200 participants is considered adequate for models with moderate complexity and few latent variables [62]. Additionally, Wolf and their colleagues [63] suggest that models with 10–20 free parameters require between 200–400 cases for sufficient statistical power and stable estimates. Our model, which included 16 parameters and a parallel mediation structure, falls within this acceptable range. The study protocol was reviewed and approved by the Institutional Review Board of the University of South Carolina.

### Measures

#### Demographic variables

Our survey contained questions that captured key demographic variables, such as age, sex, race, and education level. Participants were asked to identify their sex as Male, Female, or Other. Race was categorized into White, Black/African American, Hispanic/Latino, and Other. Education was recorded at various levels, from less than a high school diploma to a post-graduate degree, to understand educational attainment. Socioeconomic variables, namely employment and caretaker status, were also gathered to provide context for the participants' living conditions and responsibilities. Employment status was detailed in categories ranging from full-time in-person and remote work to part-time employment, unemployment, and other statuses such as retirement. Lastly, caretaker status ranged from sole caretakers to those sharing responsibilities and non-caretakers. This binary variable assigns a value of 0 to participants who are either not the sole caretaker of their family, share caregiving responsibilities with someone else, or were not involved with caretaker responsibilities. It assigns a value of 1 to those who are the sole caretaker, or even if they had partners, their partners did not assume any caregiving responsibilities.

#### Social Support

A structured scale based on the Modified Medical Outcomes Study Social Support Survey (mMOS-SS) 8-item scale [64] was administered to measure social support, asking participants how often they receive support from various sources when needed, including family members (e.g., parents, siblings), spouse or partner, friends, colleagues/co-workers, neighbors, church members, healthcare providers, and others. Responses were captured on a 6-point scale: "Not applicable," "Never," "Rarely," "Sometimes," "Pretty often," and "A lot." Higher scores on this scale indicate more frequent support from these social connections. The scale showed good reliability in this study with a Cronbach's alpha of 0.85.

#### Resilience

The Brief Resilience Scale (BRS) was used to measure resilience. BRS, developed by Smith et al. [65], is designed to assess an individual’s ability to recover from stress and adversity. This scale consists of six items, scored on a 5-point Likert scale, ranging from 1 (“Strongly Disagree”) to 5 (“Strongly Agree”). BRS includes some positively worded items, such as “I tend to bounce back quickly after hard times,” and some negatively worded items, such as “I have a hard time making it through stressful events.” To get an overall resilience score, scores from negatively worded items were reversed. Then, an average of all scores indicates the resilience score, with higher values indicating greater resilience. Reliability analysis for the Resilience scale showed a Cronbach’s alpha of 0.73.

#### Coping

A subset of 13 items from the Brief COPE scale was used in our study, developed by Carver [66], as a tool for assessing various coping strategies. Reliability analysis for the Coping scale showed a Cronbach's alpha of 0.83. This study grouped coping mechanisms into two different groups, positive coping and negative coping.

##### Positive Coping

This item is the sum of the items that address stressful events healthily and constructively. It was measured through items focused on addressing problems, emotional support, professional help, positive reframing, humor, acceptance, and religion. Reliability analysis for this subscale showed a Cronbach's alpha of 0.82.

##### Negative Coping

This item is the sum of the items that address stressful events using strategies that are hurtful to both physical and mental health in the long term. This was measured using items focused on distraction, denial, substance use, giving up, venting, and self-blame. Reliability analysis for this subscale showed a Cronbach's alpha of 0.77.

#### Post-Traumatic Stress Disorder (PTSD)

We adapted a subset of five items from the PTSD Symptom Scale-Self Report for DSM-5 (PSS-SR5), originally developed by Foa [67], to assess PTSD-related symptoms experienced by participants over the past four weeks. The adapted items captured core aspects of PTSD symptomatology, including intrusive thoughts or nightmares related to the traumatic event, efforts to avoid reminders of the event, heightened vigilance or startle response, emotional detachment from people and activities, and feelings of guilt or self-blame associated with the event. Participants were asked to respond "yes" or "no" to each item, indicating whether they had experienced each symptom. Composite scores for PTSD were calculated by summing the number of positive responses to the symptoms. The PTSD measure used in our study demonstrated strong reliability with a Cronbach's alpha of 0.84.

#### Hopelessness

To measure hopelessness, we utilized the Beck Hopelessness Scale (BHS), developed by Beck [68]. The scale included 20 items, asking participants to respond to each statement with "True" or "False" based on their feelings over the past week. We calculated the composite scores by summing the scores across all items and assigning 1 point for responses that indicate hopelessness. Scores on this scale range from 0 to 20, with higher scores indicating greater hopelessness. The hopelessness level is categorized as minimal (0–3), mild (4–8), moderate (9–14), and severe (15–20) on this scale. The Hopelessness scale used in our research exhibited high reliability, with a Cronbach’s alpha of 0.85.

### Data Analysis

#### Missing Data

We used mode imputation to handle the missing data for categorical variables in our survey responses. This method is straightforward and one of the fastest methods to deal with missing data in categorical variables [69, 70]. In our datasets, we did not use any imputations for continuous variables (Social support, negative/positive coping, resilience, PTSD, and hopelessness) because all our composite scores were constructed from individual items that were categorical and were imputed. While age, the other continuous variable did not have any missing data.

#### Descriptive Analysis

For the descriptive analysis, we examined the demographic characteristics of the sample, including age, education level, annual income, employment status, and household size. We calculated the frequency and percentage for categorical variables, such as education, income, and employment status. We reported the mean and standard deviation for age as a continuous variable.

#### Path Analysis

Our SEM model tested the hypothesized relationships in which social support influenced PTSD and hopelessness through resilience and coping mechanisms by utilizing the SEM package “lavaan”. The model also incorporated moderation effects of age and caretaker status. All variables used in SEM analyses were standardized using the standardized = TRUE function in the lavaan package. This standardization allowed for comparability across path coefficients and improved the interpretability of the results. We first conducted a Pearson correlation analysis among the variables to gain an overall understanding of their associations so we could make informed decisions about our paths in our final model. To do this, we used R programming language and the “psych” package. In our model, we specified the direct effects of social support on negative and positive coping, resilience, and hopelessness with PTSD. Indirect effects were calculated to capture mediated pathways, such as the effect of social support on PTSD through resilience and negative and positive coping. We also defined total effects to assess both the direct and mediation effects of social support on PTSD and hopelessness. Total effects were computed as the sum of all direct and indirect effects along the defined paths using the following formula: c′ + (a × b). Indirect effects represent the influence of social support on outcomes that were transmitted through mediating variables (e.g., resilience, coping) and were calculated by multiplying the path from the independent variable to the mediator (a) by the path from the mediator to the outcome (b). These provide an estimate of the overall impact of social support on mental health outcomes, accounting for multiple mediating mechanisms. Using Maximum Likelihood estimation with bootstrap standard errors (1,000 samples), we summarized model fit and path coefficients, including standardized estimates, to evaluate the significance and strength of each relationship in our model. In addition, we have added a covariance between negative and positive coping variables as both were from the same higher-order construct and scale, which was coping, so we could account for their shared variances.

## Results

The participants (n = 328) had a mean age of 48.03 years and an SD of 16.75. Regarding caretaker status, 23.2% were not the sole caretaker, 40.2% took full responsibility for their households, some shared caretaking duties (27.4%), 4.3% had a partner who did not share any responsibility, and 4.9% of the participants did not report their caretaker status. Employment status among the participants varied, most of them were working full-time in-person or remotely (48.8%), 15.2% were retired, and others were engaged in part-time work (11.8%), self-employment (2.4%), or unemployed (8.2%), disabled (4.9%), others (homemaker, and maternity/sick leave) accounted for 6.3%, and 2.1% not reported. Educational backgrounds also differed, ranging from less than a high school diploma to post-graduate degrees, the most common being a high school diploma (26.2%) or some college but no degree (22%), as shown in Table 1.

**Table 1:** Demographic and Socioeconomic Profile of Study Participants

Category	Frequency (N)	Percentage (%)
Caretaker Status		
Not the sole caretaker	76	23.2
Sole caretaker	132	40.2
Shares caretaking responsibilities with a Partner	90	27.4
Has a partner who does not share the responsibilities	14	4.3
Total	312	95.1
Not reported/Missing	16	4.9
Total	328	100
Employment Status		
Working full-time in-person	117	35.7
Working full-time remote	43	13.1
Working part-time in-person	29	8.8
Working part-time remote	10	3.0
Self-employed	8	2.4
Unemployed – looking for work	19	5.8
Unemployed – not looking for work	8	2.4
Retired	50	15.2
Homemaker	16	4.8
Maternity leave	1	0.3
Illness/sick leave	4	1.2
Disabled	16	4.9
Total	321	97.9
Not reported/Missing	7	2.1
Total	328	100
Education Level		
Less than a high school diploma	14	4.3
High school degree or equivalent (GED)	86	26.2
Some college, but no degree	72	22.0
Associate degree (AA, AS)	51	15.5
Bachelor’s degree (BA, BS)	59	18.0
Post-graduate degree or above (Master’s, PhD, MD, JD)	37	11.3
Total	319	97.3
Not reported/Missing	9	2.7
Total	328	100

### Pearson Correlation

As shown in Table 2, the correlation analysis revealed that the strongest relationships were between resilience and hopelessness (*r* = −0.553) and negative coping and PTSD (*r* = 0.418). Resilience showed significant negative relationships with both PTSD (*r* = −0.235) and negative coping (*r* = −0.359). PTSD and positive coping were both significantly correlated with hopelessness, with correlation coefficients of *r* = −0.17 and *r* = 0.38, respectively. Lastly, social support showed negative significant correlations with hopelessness (*r* = −0.284), PTSD (*r* = −0.205), and negative (*r* = −0.199) and positive coping (*r* = −0.183).

**Table 2:** Correlation Matrix of Social Support, Coping, Resilience, PTSD, and Hopelessness

Variable	Social	Resilience	Hopelessness	PTSD	Positive Coping	Negative Coping
Social	1					
Resilience	0.302^**^	1				
Hopelessness	−0.284^**^	−0.553^**^	1			
PTSD	−0.205^**^	−0.235^**^	0.178^**^	1		
Positive Coping	−0.183^**^	0.017	−0.17^**^	0.212^**^	1	
Negative Coping	−0.199^**^	−0.359^**^	0.319^**^	0.418^**^	0.489^**^	1

^**^ Correlation is significant at the 0.01 level

^*^ Correlation is significant at the 0.05 level

### Direct Effect Analysis

**Fig. 2:**
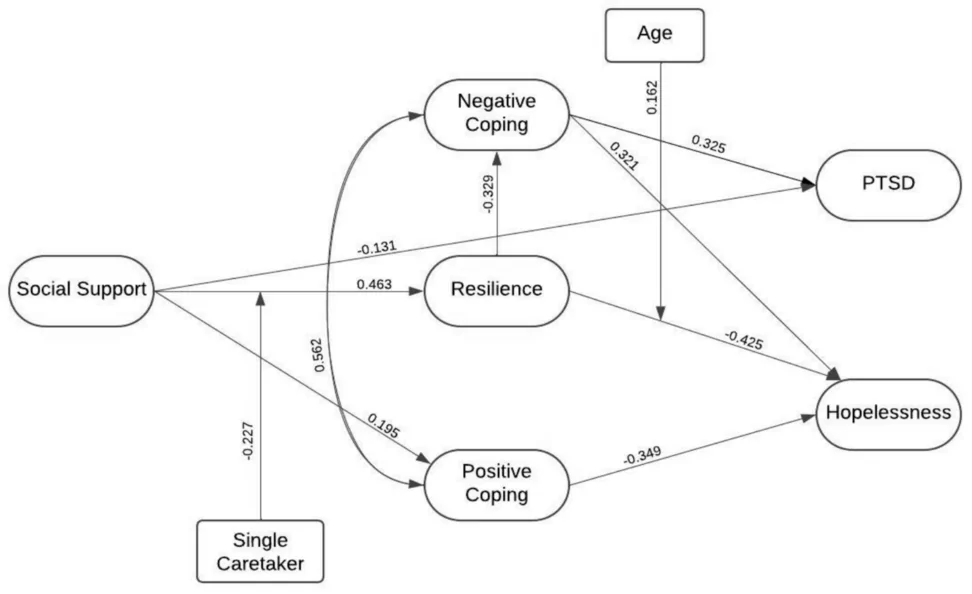
Path Model of Direct Effect among Social Support, Coping, Resilience, PTSD, and Hopelessness

Path analysis revealed an overall acceptable fit index (*CFI* = 0.99, *TLI* = 0.98, *RMSEA* = 0.034, *SRMR* = 0.031) with a *chi-square* of 11.02 (*p-value* = 0.2), suggesting a good fit between our proposed model and the observed data (see Fig. 2).

As shown in Table 3, social support had a significant positive association with Resilience (β = 0.463, *p* < 0.001) among African American rural women. While resilience was negatively associated with negative coping (*β* = −0.329, *p* < 0.001), it did not show any associations with positive coping. Additionally, the analysis showed that those with social support were more likely to utilize positive coping strategies (*β* = 0.195, *p* = 0.001). However, social support did not show any significant association with negative coping among the sample. Among predictors of PTSD among African American rural women, the largest effect size was for negative coping (*β* = 0.325, *p* < 0.001), while social support was also significantly associated with PTSD (*β* = −0.131, *p* = 0.042). On the other hand, resilience and positive coping did not show significant associations with PTSD.

**Table 3:** Summary of Direct Effects of Social Support, Coping, Resilience, PTSD, and Hopelessness

Effect	*β*	*Se*	*P value*	*Ci Upper*	*Ci Lower*
Social Support → Positive Coping	0.195	0.043	0.001	0.062	0.225
Resilience → Positive Coping	−0.042	0.579	0.504	−1.557	0.699
Social Support → Negative Coping	−0.099	0.026	0.08	−0.098	0.002
Resilience → Negative Coping	−0.329	0.319	< 0.001	−2.441	−1.17
Resilience → PTSD	−0.081	0.128	0.181	−0.41	0.075
Negative Coping → PTSD	0.325	0.029	< 0.001	0.068	0.182
Social Support → PTSD	−0.131	0.011	0.042	−0.046	−0.002
Positive Coping → PTSD	0.078	0.014	0.191	−0.01	0.045
Resilience → Hopelessness	−0.425	0.228	< 0.000	−2.486	−1.597
Social Support → Hopelessness	−0.016	0.018	0.731	−0.043	0.029
Negative Coping → Hopelessness	0.321	0.053	< 0.001	0.165	0.371
Positive Coping → Hopelessness	−0.349	0.027	< 0.000	−0.236	−0.128
Social Support → Resilience	0.463	0.007	< 0.001	0.025	0.051
Social Support * Caretaker → Resilience	−0.227	0.01	0.006	−0.045	−0.008
Resilience * Age → Hopelessness	0.162	0.236	< 0.001	0.462	1.36

Regarding hopelessness, resilience again showed to play a key role for African American rural women as it had a strong positive significant direct effect on hopelessness (*β* = −0.425, *p* < 0.000). Also, positive coping had a negative significant effect (*β* = −0.349, *p* < 0.000) on hopelessness. Like PTSD, negative coping had a positive significant association with hopelessness (*β* = 0.321, *p* < 0.001). Social support did not show any significant effect on hopelessness.

The moderation analysis, shown in Table 3, demonstrated that single caretaker status negatively moderated the association between social support and resilience (β = −0.227, p = 0.006), while age positively moderated the association between resilience and hopelessness (β = 0.162, p = 0.001). The protective effect of social support is stronger among older women but weaker among single caretakers.

### Indirect/Mediation Effects

As shown in Table 4, the indirect effect of social support on PTSD through resilience was not significant, meaning resilience among African American rural women still did not improve the social support effect on PTSD. However, its indirect effect on hopelessness through resilience was negatively significant (*β* = −0.197, *p* =  < 0.000). Suggesting the mediating role of resilience on the association between social support and hopelessness, which was initially non-significant. Social support's indirect effect on both PTSD and Hopelessness was not significant through negative coping. However, its indirect effect on hopelessness through positive coping was significant (*β* = −0.068, *p* = 0.002), suggesting a mediating role of positive coping strategies in the association between social support and hopelessness among African American rural women.

**Table 4:** Summary of Mediation/Indirect Effects among Social Support, Coping, Resilience, PTSD, and Hopelessness

Effect	*β*	*Se*	*P value*	*Ci Lower*	*Ci Upper*
Social Support → Resilience → PTSD	−0.037	0.005	0.208	−0.017	0.003
Social Support → Resilience → Hopelessness	−0.197	0.015	< 0.000	−0.109	−0.049
Social Support → Negative Coping → PTSD	−0.032	0.003	0.114	−0.013	0.00
Social Support → Negative Coping → Hopelessness	−0.032	0.008	0.104	−0.029	0.00
Social Support → Positive Coping → PTSD	0.015	0.002	0.246	−0.001	0.008
Social Support → Positive Coping → Hopelessness	−0.068	0.008	0.002	−0.043	−0.011

### Total Effects

Regarding total effects, as shown in Table 5, both effects of social support on PTSD and hopelessness through resilience were significant with coefficients of *β* = −0.168, *p* = 0.007, and *β* = −0.213, *p* < 0.000, respectively. The total effect of social support on PTSD through negative coping was significant *β* = −0.163, *p* = 0.014, suggesting that social support among African American rural women is associated with lower levels of PTSD even if they use negative coping strategies. All other total effects did not show significant associations, such as the social support effect on PTSD and hopelessness through positive coping.

**Table 5:** Summary of Total Effects in the Model

Total Effect	*β*	*Se*	*P value*	*Ci Lower*	*Ci Upper*
Social Support → Resilience → PTSD	−0.168	0.011	0.007	−0.051	−0.009
Social Support → Resilience → Hopelessness	−0.213	0.021	< 0.000	−0.124	−0.041
Social Support → Negative Coping → PTSD	−0.163	0.011	0.014	−0.052	−0.007
Social Support → Negative Coping → Hopelessness	−0.048	0.018	0.297	−0.054	0.015
Social Support → Positive Coping → PTSD	−0.116	0.011	0.068	−0.043	−0.001
Social Support → Positive Coping → Hopelessness	−0.085	0.02	0.099	−0.07	0.006

## Discussion

This study examined how social support relates to psychological distress, specifically PTSD and hopelessness, among African American women in rural SC, during the COVID-19 pandemic. Findings revealed that these relationships were significantly mediated by resilience and coping strategies and moderated by age and caregiving status.

Our results indicate that social support significantly reduced PTSD symptoms but did not have a direct impact on hopelessness. This finding aligns with previous research suggesting that social support can buffer the effects of trauma by providing emotional, informational, and tangible resources that promote psychological well-being [36, 71]. African American women in rural areas, who often face structural barriers to mental health care, may rely more heavily on informal support networks such as family, church communities, and community organizations for crisis management [26]. However, the lack of an association between social support and hopelessness suggests that while social support may mitigate trauma-related symptoms, it does not necessarily instill hope or a sense of optimism about the future, especially with the uncertainty for the future caused by COVID-19. This aligns with research indicating that chronic stressors such as systemic racism, economic hardship, and caretaker burdens may contribute to sustained hopelessness despite external support [7]. Therefore, community-based interventions should prioritize strengthening social networks through culturally tailored mental health programs, ensuring that these supports extend beyond crisis periods for long-term psychological resilience.

Consistent with previous studies, resilience emerged as a key protective factor against hopelessness during the COVID-19 pandemic, reinforcing the notion that personal adaptive capacity is critical for maintaining psychological well-being in the face of adversity [18]. However, a noteworthy and somewhat counterintuitive finding was the positive association between resilience and PTSD among aging African American women. This suggests that while resilience generally promotes psychological adaptation, its protective effects may diminish with age, potentially due to cumulative exposure to stress and trauma and managing comorbid conditions in the COVID-19 pandemic. Older African American women in rural SC often bear the compounded burden of lifetime racial discrimination, caregiving responsibilities, and limited access to healthcare, which may contribute to persistent PTSD symptoms despite high resilience [20, 72]. This highlights that resilience-building alone may not be sufficient; instead, trauma-informed care models and culturally responsive interventions, are essential in addressing cumulative stress and systemic inequalities.

Positive coping strategies, such as reframing stressors and problem-solving, were associated with lower levels of hopelessness, while negative coping strategies, such as avoidance and emotional disengagement, were strongly associated with both PTSD and hopelessness. This is consistent with existing literature, which suggests that African American women often resort to both adaptive and maladaptive coping mechanisms in response to chronic stress and systemic inequities [48].

Another notable finding was that social support was associated with increased use of positive coping strategies but did not significantly reduce negative coping behaviors. This suggests that while social support encourages healthy coping for challenges during the COVID-19 pandemic, it does not necessarily prevent the use of maladaptive strategies. It seems that many African American women, particularly those facing structural barriers such as poverty, healthcare inaccessibility, and systemic discrimination, may turn to negative coping mechanisms out of necessity rather than choice [26]. Strategies such as emotional suppression, avoidance, and distraction can serve as temporary survival tactics in the face of ongoing economic and social stressors, yet these methods become maladaptive when used as long-term coping mechanisms.

One of the most striking findings was the negative association between social support and resilience among single caretakers. This suggests that during the COVID-19 pandemic, for African American women who are the sole caretakers of their families, social support may not translate into increased resilience but may instead reflect dependency or an overreliance on external resources. Previous research has suggested that African American women, due to cultural expectations and the SBW construct, may feel a responsibility to manage hardships independently [4]. When these women receive social support, it may not necessarily empower them but rather highlight their reliance on external help, potentially leading to a perceived loss of self-sufficiency, especially in crisis management. This underscores the importance of designing social support interventions that not only provide external resources but also empower women with skills and opportunities to strengthen their own resilience and self-efficacy, such as financial literacy training, childcare support, job training, and community mentorship programs.

While this study provides valuable insights into the mental health outcomes of rural African American women in SC during the COVID-19 pandemic, several limitations should be acknowledged. First, the cross-sectional design restricts the ability to draw causal inferences. Although SEM allows for testing theoretically informed pathways, it does not confirm temporal sequencing. It is plausible, for instance, that individuals experiencing psychological distress may be more likely to seek social support or develop coping mechanisms in response. Likewise, resilience may emerge as a reaction to adversity rather than functioning exclusively as a protective factor. These possibilities underscore the need for longitudinal studies to establish the directionality and temporal ordering among social support, coping, resilience, and psychological distress. Second, data were collected between January and February 2024, a period reflecting the post-pandemic recovery phase rather than the acute phase of the COVID-19 crisis. Although participants were asked to retrospectively assess their pandemic-related distress, recall bias may have influenced their responses. Future research should consider real-time or longitudinal data collection to better capture the evolving nature of psychological distress. Third, the study relied exclusively on self-report measures. While these provide essential subjective insights, they are subject to potential biases such as social desirability or misreporting. Incorporating objective indicators of stress, such as cortisol levels or heart rate variability, could enhance validity in future studies. Fourth, the sample was skewed toward individuals with higher levels of educational attainment, which may limit the generalizability of findings to populations with lower educational backgrounds. Efforts to recruit a more socioeconomically diverse sample would strengthen external validity. Fifth, while marital or partner status may be a meaningful element of social support, it was not included in our analysis in order to maintain model parsimony and limit potential multicollinearity. Nevertheless, future studies should explore relationship status more explicitly as it may influence access to social and emotional support in ways that shape psychological resilience and distress. Finally, although this study focused on African American women in rural SC, its findings may not fully extend to other geographic regions or populations. Future research should explore how contextual factors, such as healthcare access, economic recovery, and community resilience, differ across rural areas in the Southern US. and beyond to provide a more comprehensive understanding of mental health disparities.

These findings have important implications for the development of tailored, community-based interventions to address mental health disparities among rural African American women. First, given the protective role of social support and resilience, interventions should aim to strengthen both formal and informal support systems. Community mental health programs could incorporate peer mentoring, group-based support, and faith-based partnerships that resonate with cultural values and local infrastructure. Second, the differential impact of age and caregiving status on the effectiveness of social support highlights the need for personalized approaches. For example, sole caregivers may benefit more from programs that integrate practical resources such as respite care, financial assistance, and parenting support, in addition to emotional or psychological counseling. Older women, meanwhile, may require interventions that address accumulated stress and trauma through trauma-informed and age-sensitive modalities. Third, the findings underscore the importance of integrating psychological resilience training into routine health services, including primary care settings, especially in medically underserved areas. Such training may include cognitive-behavioral strategies, stress appraisal techniques, or culturally tailored coping workshops. Finally, to address systemic contributors to distress, broader policy-level actions such as expanding Medicaid coverage, improving access to mental health professionals in rural areas, and funding local infrastructure to support caregiver networks are needed. This multilevel approach is critical to advancing health equity and ensuring that mental health services meet the needs of vulnerable populations.

## Conclusion

This study underscores the nuanced relationship among social support, resilience, coping, and mental health outcomes among rural African American women in SC. While social support plays a crucial role in mitigating PTSD, its effects on hopelessness are mediated through resilience and positive coping strategies. However, age and caretaker status significantly moderate these relationships, indicating that a one-size-fits-all approach to mental health interventions may be insufficient. Future efforts should prioritize tailored, culturally responsive interventions that address the specific needs of aging women, sole caretakers, and those at risk of negative coping behaviors. By adopting a holistic, community-engaged approach, mental health professionals and policymakers can develop more effective strategies to promote psychological well-being in this vulnerable yet resilient population.

## Data Availability

Data is available from the corresponding author upon reasonable request.
